# The Danger of Posterior Plagiocephaly

**Published:** 2015-05-12

**Authors:** Susan Orra, Kashyap Komarraju Tadisina, Bahar Bassiri Gharb, Antonio Rampazzo, Gaby Doumit, Francis Papay

**Affiliations:** ^a^Department of Plastic Surgery, Cleveland Clinic, Cleveland, Ohio; ^b^Cleveland Clinic Lerner College of Medicine of Case Western Reserve University, Cleveland, Ohio

**Keywords:** posterior plagiocephaly, lambdoid craniosynostosis, deformational plagiocephaly, Back to Sleep campaign, distraction osteogenesis

## DESCRIPTION

A 17-month-old patient was referred to the pediatric plastic surgery clinic for asymmetrical head shape and left torticollis. On examination, asymmetry of the occiput was noted and a diagnosis of posterior plagiocephaly was made. Helmet therapy was not recommended because it is not effective for patients older than 18 months. The patient was referred to obtain radiographs and computed tomographic (CT) scan of the skull to rule out possible lambdoid craniosynostosis and told to continue physical therapy. Review of CT scans indicated right lambdoid suture closure. Neurosurgical evaluation yielded increased intracranial pressure, and distraction osteogenesis of the posterior cranial vault was performed for the duration of 2 months without complications. Head symmetry improved, and the patient is doing well postoperatively.

## QUESTIONS

1. **What is the concern when patients present with posterior plagiocephaly?**
2. **How has the incidence of posterior plagiocephaly changed with the “Back to Sleep” campaign?**
3. **What is the diagnostic workup protocol for these patients?**
4. **Describe the conservative and surgical management protocols for these patients.**

## DISCUSSION

“Posterior plagiocephaly” is a term used to describe an asymmetry in the posterior cranial vault of the head. It can be “deformational plagiocephaly,” in which extrinsic forces mold the head into an asymmetrical shape, or it can be a consequence of “lambdoid craniosynostosis,” or premature fusion of the lambdoid suture due to intrinsic factors. Lambdoid craniosynostosis is a rare condition affecting less than 2% of infants, making it the rarest of all types of craniosynostosis. If left untreated, it can lead to developmental delay, mental retardation, and inhibitory growth of the cerebrum. Although historically thought to be only a “cosmetic” concern, deformational plagiocephaly has recently been implicated in causing developmental changes in the brain. Collett et al^1^ compared craniosynostotic plagiocephaly with deformational plagiocephaly and confirmed that infants were just as likely to suffer developmental delays from suture fusion as from positional molding. A recent study of school-aged patients determined that children with deformational plagiocephaly were more likely to require special education services in school including speech therapy, occupational therapy, and physical therapy.^2^ Hence, regardless of the cause, it is important to screen for posterior plagiocephaly early in infancy so that appropriate treatment can be implemented.

The “Back to Sleep” campaign was implemented in 1992 to reduce the incidence of sudden infant death syndrome by recommending that parents put their infants to sleep in the supine position rather than the prone position. In parallel, the incidence of posterior deformational plagiocephaly has substantially increased. Prior to the “Back to Sleep” campaign, the incidence was estimated to be 1 in 300 infants (0.3%). Current incidence is estimated to be as high as 8.2%.^3^ The sharp rise in deformational plagiocephaly has also resulted in a false rise in the rate of diagnosis of craniosynostotic plagiocephaly. Inadequate knowledge of physical features and the complicated process of differentiating the 2 disease entities among pediatricians have resulted in unnecessary referrals and delays in treating infants. Given that the treatment of deformational plagiocephaly is generally conservative and lambdoid synostotic plagiocephaly requires surgery, it is imperative to differentiate these 2 causes.

Both lambdoid craniosynostosis and deformational plagiocephaly are ideally diagnosed within the first 18 months of life,^4^ although the majority of synostotic plagiocephaly cases are often diagnosed at birth or within the first 3 to 6 months of life. On physical examination, lambdoid craniosynostosis has a trapezoid shape when viewed from above and the ipsilateral ear is displaced posteriorly and inferiorly. A bony ridge is palpable over the closed lambdoid suture, and there is unilateral occipitoparietal flattening posteriorly. Posteriorly, there is an ipsilateral occipitomastoid bulge and the skull base appears tilted. Diagnostic imaging is usually performed to confirm physical examination findings and includes a head CT scan with 3-dimensional reconstruction, CT scan with bone windows, and/or Towne's view.^5^^,^^6^ In contrast, deformational plagiocephaly is not present at birth. Characteristic physical examination findings include a parallelogram when viewed from above, ipsilateral ear displaced anteriorly, and no bony ridge over the lambdoid suture. It usually presents as unilateral occipitoparietal flattening but can be bilateral. Finally, when viewed posteriorly, the skull base is horizontal and no occipitomastoid bulge is present. Computed tomographic and radiographic evidence usually reveals open sutures, and the infant may have torticollis of the sternocleidomastoid muscle.^6^

Posterior plagiocephaly is often managed conservatively before consideration of any surgical intervention. Conservative management includes helmet therapy and repositioning. Optimal age for starting helmet therapy is 5 to 6 months of life, and it has not been found to be effective after 18 months of age.^7^ Complete resolution of deformational plagiocephaly with helmet therapy has been reported in 60% to 90% of cases, with even 100% of patients improving in appearance by 4 months in 1 study.^3^ Helmet therapy was more effective in correcting deformational plagiocephaly than repositioning alone, although avoiding long periods in a car seat, physical therapy for infants with torticollis, tummy time, and turning the head regularly have all been found to be helpful.^8^ Surgery is the first-line intervention in the case of true lambdoid craniosynostosis. Correction is aimed to be completed within the first year of life to optimize brain development and take advantage of the malleability of cranial bones. The major operative risk relates to blood loss, with even small amounts of blood loss being meaningful in very young children. This has given impetus to the rebirth of “strip craniectomy” procedures, which use endoscopic technique and postoperative skull molding caps. The essential features of treating craniosynostosis are release of the fused suture and remodeling of both hypoplastic and compensatory growth abnormalities. Children who had uncorrected craniosynostosis for periods longer than 1 year showed significantly reduced intelligence quotients when compared with patients who had surgery before 1 year of age.^4^

Posterior plagiocephaly is a condition that most craniofacial and pediatric plastic surgeons are bound to encounter in their practice, particularly since the “Back to Sleep” campaign was instituted. A proactive approach to screening these patients is recommended to uncover whether deformational plagiocephaly or lambdoid craniosynostosis is the true cause. Each condition has distinct physical examination features and dichotomous treatment approaches, conservative (deformational plagiocephaly) or invasive (lambdoid craniosynostosis). Literature is replete with evidence that lambdoid craniosynostosis is associated with developmental delays. In addition, recent studies have demonstrated that even children who had deformational plagiocephaly during infancy had more difficulty in school later in life than their counterparts, making aggressive screening for children who present with asymmetric head shape of paramount importance.

## Figures and Tables

**Figure 1:**
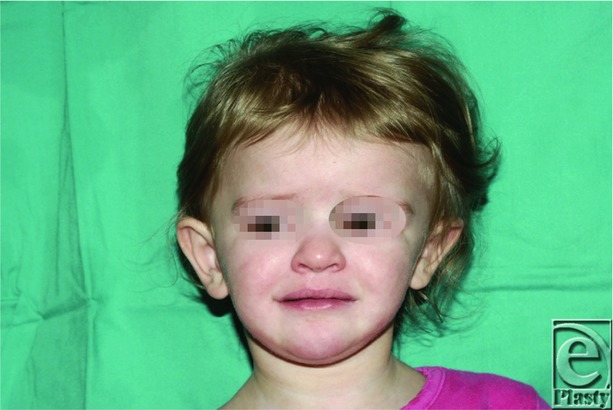
Preoperative at 22 months old. Asymmetry of the cranium noted in which the cranium is growing superiorly and laterally on the left side of the patient's head due to a fused right lambdoid suture.

**Figure 2:**
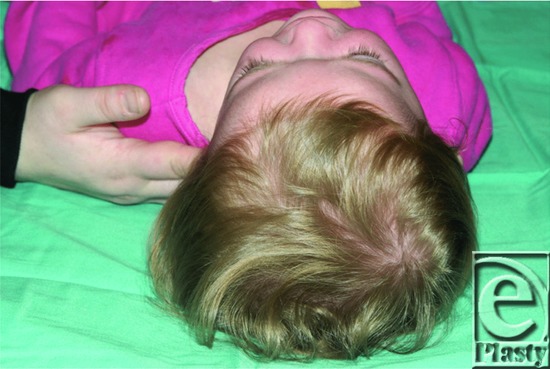
Preoperative photograph showing a superior view demonstrating asymmetrical cranial growth of the left side of the patient's head due to a fused lambdoid suture on the right.

**Figure 3:**
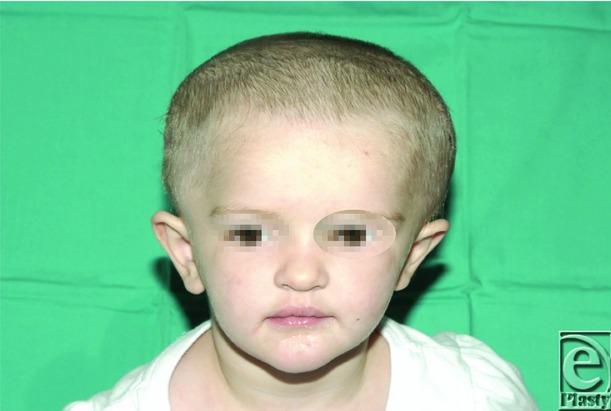
Four months postoperative after distraction osteogenesis of the posterior cranial vault (distraction osteogenesis performed for the duration of 2 months).

**Figure 4:**
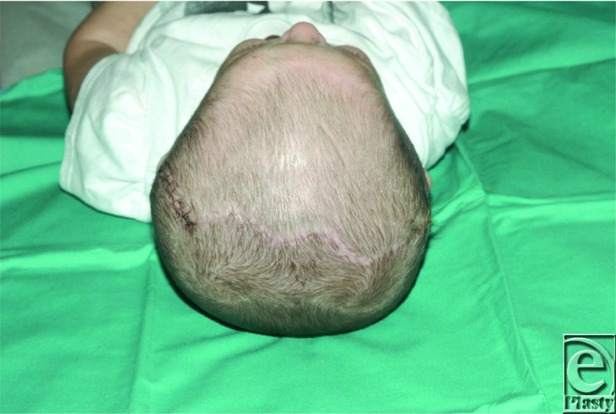
Photograph demonstrating the incision pattern after 2 months of distraction osteogenesis of the posterior cranial vault.
